# Validity and reliability of a novel Color-Risk Psychiatric Triage in a psychiatric emergency department

**DOI:** 10.1186/s12888-016-0727-7

**Published:** 2016-02-10

**Authors:** Alejandro Molina-López, Jeremy Bernardo Cruz-Islas, Mauricio Palma-Cortés, Diana Patricia Guizar-Sánchez, César Yehú Garfias-Rau, Martha Patricia Ontiveros-Uribe, Ana Fresán-Orellana

**Affiliations:** Continuous Psychiatric Care Department, Clinical Services Direction, Ramon de la Fuente National Institute of Psychiatry, Calz. México-Xochimilco 101, Mexico City, 14370 Mexico; Education Direction, Ramon de la Fuente National Institute of Psychiatry, Mexico City, Mexico; Clinical Services Direction, Ramon de la Fuente National Institute of Psychiatry, Mexico City, Mexico; Clinical Epidemiology Laboratory, Clinical Research Direction, Ramon de la Fuente National Institute of Psychiatry, Mexico City, Mexico

**Keywords:** Triage, Psychiatric emergency presentations, Classification, Emergency department

## Abstract

**Background:**

Classification of Psychiatric Emergency Presentations (PEP) is not sufficiently clear due to their inherent high inter-subjectivity and lack of validated triage instruments. In order to improve current classification of psychiatric emergency presentations (PEP) at Emergency Departments, we implemented and validated the Color-Risk Psychiatric Triage (CRPT), an instrument for classifying PEP risk by sorting one to five color/risk levels and one to thirty-two possible conditions arranged by risk.

**Methods:**

Users who visited the Emergency Department (ED) of a Mexican psychiatric hospital from Dec 1st, 2008 to Dec 1st, 2009 were included. One CRPT was assessed by an ED psychiatrist to each patient upon their arrival to ED. Some patients were randomly assessed simultaneously with an additional CRPT and a Crisis and Triage Rating Scale (CTRS) to test validity and reliability of the CRPT.

**Results:**

A total of 7,631 CRPT assessments were included. The majority of PEP were non-urgent (74.28 %). For the validation phase *n* = 158 patients were included. CRPT score showed higher concurrent validity than CRPT color/risk. CRPT level/risk and score showed highest concurrent validity within dangerousness domain of CTRS (*r* = 0.703, *p* < 0.0001). CRPT and CTRS scores showed similar predictive validity (*p* < 0.0001). High intraclass correlation coefficient (0.982) and Cohen’s Kappa (0.89) were observed for CRPT score (*r* = 0.982, *p* < 0.0001).

**Conclusions:**

CRPT appeared to be a useful instrument for PEP classification due to its concurrent validity, predictive validity and reliability. CRPT score showed higher correlations than the CRPT color/risk. The five levels of risk provided by the CRPT appear to represent a simple and specific method for classifying PEP. This approach considers actual or potential risk, rather than severity, as the main factor for sorting PEP, which improves upon the current approach to emergency classification that is mainly based on the criterion of severity. Regardless of the triage procedure, emergency assessments should no longer classify PEP as “not real emergencies.”

**Electronic supplementary material:**

The online version of this article (doi:10.1186/s12888-016-0727-7) contains supplementary material, which is available to authorized users.

## Background

In the last few decades, utilization of psychiatric emergency services (PES) has increased in many countries around the world [1–3]. Factors such as deinstitutionalization [4], poor accessibility to specialized services, the need for more continuous provision of care [5], increasing populations [6], use of alcohol and other substances [7], and increased suicidality [8] have contributed to the enhanced demand for PES.

To face this increased demand, medical and psychiatric health systems have made improvements to the administrative and quality procedures employed in cases of psychiatric emergencies. One such procedure is the classification of psychiatric emergency presentations (PEP). PEP represent a wide range of heterogeneous and complex conditions that are difficult to classify because of the high inter-subjectivity and lack of systematic methods for identifying psychiatric emergencies, both at general and psychiatric hospitals [9]. Some authors have even suggested that inter-subjectivity in psychiatry should involve a level of integration between social perception and self-perception [10].

Descriptive data about PEP in certain populations often reveal the existence of an acute need for immediate attention, especially when outpatient services are not available [11]. To differentiate between urgent and non-urgent PEP, classifications should be made with quick, valid and reliable assessments.

Numerous efforts have been made to classify PEP. For instance, Chaput et al. [12] proposed a qualitative classification of PEP into three categories: pertinent and urgent, pertinent but not urgent, and neither pertinent nor urgent. However, the use of qualitative as opposed to structured assessments in PES may lead to inaccurate outcomes due to extremely non-specific and variable points of view on the part of both clients and physicians.

One well-accepted strategy for classifying the severity of medical emergencies is triage assessment. Triage is a French word that means ‘to sort’. Medical triage scales help to classify medical emergencies to identify those that are the most life-threatening and to estimate the maximum wait time for the first medical intervention [13]. Current medical triage scales such as the Manchester Triage [14] and the Canadian Triage and Acuity Scale [15] are based on five-color codes and are used in a wide variety of medical settings. Triage assessments should always be made at first contact in the emergency department (ED) to improve organizational flow as well as facilitate admission/discharge decisions [16].

Medical emergencies differ significantly from psychiatric emergencies because the sorting process is based on the threat to life of a particular condition and the disease-related complications. This is in contrast to psychiatric assessments, in which the focus is on assessing the level of danger presented to others or the level of severity of the impairment to social functioning. Thus, medical triage scales are not entirely suitable for sorting PEP [17]. In fact, medical triage scales often either fail to include specific criteria for the sorting of psychiatric emergencies or classify PEP as “not real emergencies”. However, there are a number of extremely dangerous PEP that can put the patient at risk of serious physical harm, which could be strictly considered non-urgent conditions by the current medical emergency criteria. Conversely, many non-urgent psychiatric conditions e.g., acute need of a medical prescription, may increase demand or even overcrowd PES. Although they are not life threatening, these presentations often stem from the users’ subjectively justified need for immediate attention from the PES.

Perhaps the most important factors when classifying PEP are the actual and potential risks, rather than features such as symptom severity. Indeed, screening for violence or suicide risk is widely recommended during triage assessment [18, 19].

There have been many efforts to design and validate mental health triage scales and instruments to sort PEP. For instance, Australian Mental Health Triages such as Hobart Mental Health Triage Scale [20] (MHTS), and South Eastern Sydney Area Health Service [21] (SESAHS), are structured classification of PEP based in medical triage procedures in Australia. MHTS and SESAHS classifies PEP into 4 and 5 categories respectively, including emergent, urgent, sub-urgent or semi-urgent and non-urgent, and assigns a maximum wait time for attending each emergency priority. Additionally, SESAHS considers observed and reported symptoms while triage assessing. Although SESAHS and MHTS has been shown to improve wait times in ED, these scales does not include medical emergency conditions and considers quite different conditions associated with PEP risks, such as violent, aggressive and suicidal behaviors, to be equal. Because aggression towards others can endanger more people than suicidal behavior, we believe that the former should be sorted into a more dangerous category than self-aggressive behaviors and non-aggressive agitation.

Another scale used for sorting PEP is the Crisis and Triage Rating Scale (CTRS), [22] which is a three-domain scale with scores ranging from 3 to 15. CTRS is designed to predict which treatment (outpatient or inpatient) is better for each patient at the time of the first contact in the ED. The CTRS has demonstrated good predictive validity, and it has been suggested that individuals with a score less than 9 should be admitted [23]. However, CTRS is not truly a risk-based PEP classification, but rather reflects a patient’s need for admission or discharge. Furthermore, CTRS does not include potentially acute medical conditions that are often comorbid with psychiatric emergencies. Another limitation of the CTRS is that it requires knowledge of each patient’s social and family support system at the time of assessment, which can be especially difficult to gauge in aggressive, agitated, suspicious, isolated or non-cooperative patients, who frequently present for PES.

Sorting PEP should be both fast and easy and should be accomplished by implementing a specific procedure that includes both medical and psychiatric emergencies. Furthermore, this approach should reduce, as much as possible, the effect of inter-subjectivity by providing a structured, inclusive assessment. Prioritizing PEP must also emphasize medical over psychiatric risk, as well as societal over individual risks. Regarding medical triage scales, we hypothesized that PEP classification could sort each actual or potential risk through a color code that suggests a maximum wait time based on the priority of each PEP.

We designed the Color-Risk Psychiatric Triage (CRPT), an instrument for sorting PEP by actual or potential risk assessments, and we tested its reliability and concurrent and predictive validity in order to improve current PEP classification.

## Methods

### Study design and settings

Study was done in Continuous Psychiatric Care Department (Previously Emergency Department) of Ramon de la Fuente National Institute of Psychiatry, which is one of the National Institutes of Health of the Mexican Federal Health Ministry, in South Mexico City. This study was approved by the Institutional Ethics Research Board of Ramon de la Fuente National Institute of Psychiatry. The implementation and standardization of the CRPT were also approved by the medical director.

### Interventions

This study was divided into two phases: a descriptive phase and a validation phase. The aim of the descriptive phase was to describe the Continuous Psychiatric Care Department’s census activity broken down by CRPT color/risk and CRPT score outcomes. The aim of the validation phase was to test the concurrent validity and reliability of the CRPT by conducting an additional CRPT assessments and one CTRS^22^ assessment simultaneously.

CRPT is shown as Additional file 1.

### Selection of participants

All users who asked for an emergency consultation at the Continuous Psychiatric Care Department from December 1st, 2008 to December 1st 30th, 2009 were included. Subjects and their relatives (if available) read and signed an informed consent form before each emergency consultation. Users younger than eighteen years had to be accompanied by at least one of their parents or guardians, and both of them (user and parent/guardian) had to accept the participation in the study and also had to sign the informed consent form. Underage users with no parents/guardian presence and/or acceptance of informed consent were not included in this study. Users were excluded if they or their relatives rejected emergency consultation. Some of the included users were randomly selected for an additional simultaneous assessment of triage procedures to test the validity and reliability of the CRPT.

### Methods and measurements

#### CRPT design

**Fig. 1:**
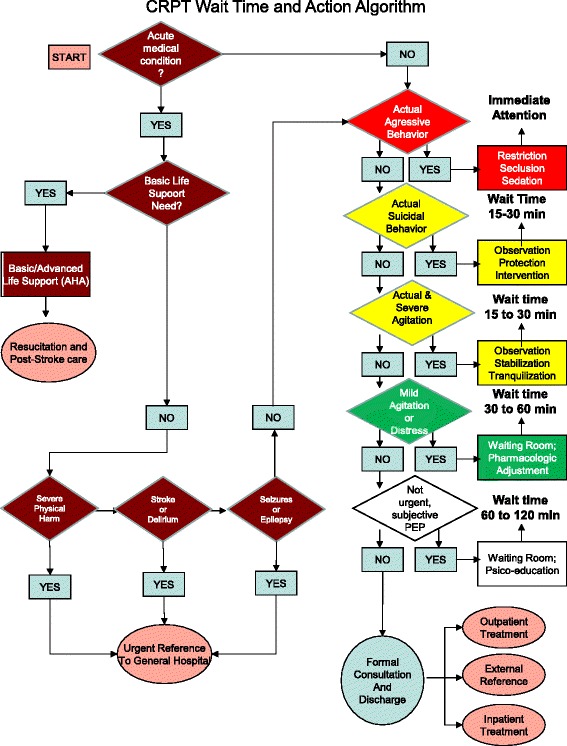
CRPT Wait Time and Action Algorithm. After CRPT assessment, each Psychiatry Emergency Presentation (PEP) had to be sort as one of five Color/Risk (Brown: Medical Emergency; Red: High Risk PEP; Yellow: Middle Risk PEP; Green: Low Risk PEP; White: No Risk PEP). It is shown the maximum recommended wait time and the possible first interventions for each Color/Risk, previously to formal consultation and discharge from ED

CRPT was designed and assessed in Spanish. The design was intended to facilitate quicker and shorter assessments for more urgent presentations, lasting only a few seconds in the most risky emergencies and extending no more three minutes in non-urgent presentations. Once the CRPT assessment was completed, each patient was seen for variable lengths of time for a first intervention and a formal consultation, as shown in Fig. 1 (CRPT Wait time and Action Algorithm). The maximum suggested wait time for each PEP after the CRPT assessment and before attendance for the PES was as follows: brown and red: immediate; yellow: 30 min; green: 60 min; and white: 120 min. The CRPT assessment and the emergency consultation were always made by different physicians.

#### Assessment staff

The CRPT was formally implemented and standardized at the CPC department on October 1st, 2008 after psychiatrists, psychiatric residents and mental health nurses had participated in several training programs. For the descriptive phase, each CRPT was assessed by either a senior psychiatrist or a fourth-year psychiatry resident. Each CRPT assessment was made as soon as a patient arrived in the CPC department. For the validation phase of the study, randomly selected patients were further simultaneously assessed by a researcher who, besides the standardized CRPT assessment, also performed a second CRPT and CTRS assessment.

#### CRPT color/risk assessment

The CRPT assessment considered two main aspects of PEP classification: color/risk and score. The goal of the CPRT color/risk was to assess the actual or potential risk level of each PEP at the time of arrival to the Continuous Psychiatric Care Department, by sorting one of the five levels of risk represented by the colors brown, red, yellow, green and white. Each color/risk was sorted by assessing each patient’s most recent and risky presentation.

#### CRPT score

The second goal of the CRPT was to determine the number-code linked to conditions that ranged from 1 to 32 and that could also be taken as a score. The 32 conditions were hierarchically ordered from the most (01) to the least (32) risky, acute and severe. When some of the 32 CRPT conditions matched with the actual condition of a current patient, the evaluator assigned a number/score and stopped the interview and any further assessment of remaining conditions. Figure 1 shows the CRPT instrument with its 5 color/risk levels and 32 conditions.

#### *Ten D’s* Mnemonics for CRPT color/risk sort

We created a *“10 D’s”* mnemonic to help sort each respective color/risk as follows: brown: medical emergency (*Delirium,* Medical *D*isease); red: high risk PEP (*D*rug Intoxication, *D*anger to Others): yellow: middle risk PEP (*D*anger to self, *D*isturbing); green: low risk PEP (*D*emand, *D*istress); and white: no risk PEP (*D*elayed, *D*etached).

#### Crisis Triage Rating Scale (CTRS) assessment

Certain randomly selected patients were further assessed with a simultaneous Spanish CTRS^22^ consisting of three domain scales: dangerousness, social support and cooperativeness assessment. The CTRS^22^ has been shown to help predict whether to admit or discharge a patient from the ED before a formal consultation. Each CTRS^22^ domain assessment consists of 5 conditions, hierarchically ordered from 1 (most severe condition) to 5 (least severe condition). Therefore, the lower the score, the higher the need to admit. Each CTRS^22^ domain has a maximum 5-point score, while the global CTRS score ranges from 3 to 15.

#### CRPT concurrent and predictive validity

To test the concurrent validity of the CRPT, we correlated the CTRS^22^ global score and each individual CTRS^22^ domain, namely dangerousness, social support and cooperativeness, with the CRPT color/risk and CRPT score. To test the predictive validity of the CRPT and CTRS^22^, we correlated clinical variables such as discharge and suicidal behavior with the CRPT risk/level and CRPT score at the end of the emergency consultation.

#### CRPT reliability

To test the reliability of the CRPT color/risk and CRPT, we compared the formal triage assessment outcomes of the CPC department staff (CRPT_1_) with a simultaneous triage assessment made by one of the researchers (CRPT_2_). CRPT_1_, CRPT_2_ and CTRS^22^ were always assessed prior to the emergency consultation. Due to dimensional characteristics of the CRPT color/risk and score, an Intra-class Correlation Coefficient (ICC) was obtained to test the CRPT’s reliability. Additionally, we used Cohen’s Kappa for testing inter-rater reliability.

### Analysis

Demographic and clinical characteristics of the descriptive phase were analyzed as categorical variables using frequencies and percentages and as continuous variables using means and standard deviations (S.D.). For CRPT validation, we used One Way ANOVA for continuous variables, and Chi Square was used for nominal variables. Spearman’s coefficient was used to correlate the CRPT level/risk and score with discharge, suicidal risk and CTRS scores. We used both Intraclass Correlation Coefficient (ICC) as well Cohen’s Kappa for testing CRPT color/risk and score inter-rater reliability. The significance level was established at *p* ≤ 0.05. The statistical software package SPSS version 22.0 for Windows P.C. was used for the data analysis.

## Results

### Characteristics of study subjects

**Fig. 2:**
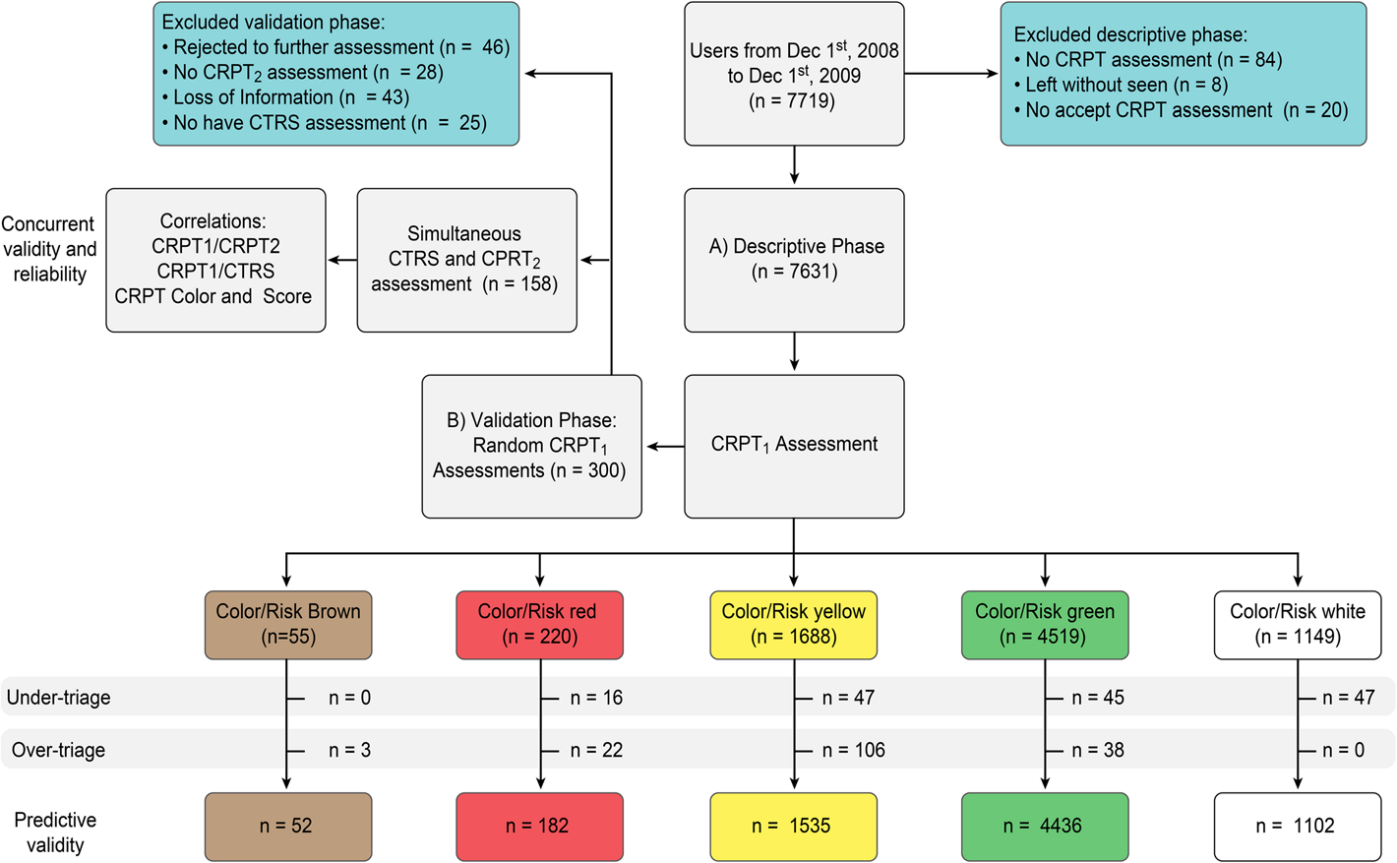
STARD Flow-Chart of descriptive and validity phases. At descriptive phase all users were assessed by the Color-Risk Psychiatric Triage (*n* = 7,631), assessed by a Psychiatrist or Psychiatry resident from the clinical staff (CRPT_1_). At validity phase a final sample of *n* = 158 random subjects were assessed by a simultaneous Color-Risk Psychiatric Triage (CRPT_2_) and a Crisis Triage Rating Scale (CTRS) in order to test reliability and concurrent validity of CRPT

A total of 7,719 PEP were registered during the descriptive phase; as shown in Fig. 2, the final sample size included 7,631 PEP. Women accounted for 67.16 % (*n* = 5,121) while men comprised 32.84 % (*n* = 2,506) of the sample. The overall mean age was 38.89 years (S.D. = 16.26). More than half the PEP included patients with a diagnosis of affective disorders (*n* = 4,373, 57.3 %), followed by anxiety and other stress-related disorders (*n* = 1,244, 16.3 %), mental disorders secondary to medical conditions (*n* = 1252, 9.1 %) and schizophrenia and other psychotic disorders (*n* = 694, 8.84 %). The remaining diagnoses represented less than 5 % of the total PEP and included personality disorders, substance-related disorders and the category of “no other specified diagnosis”.

The majority of PEP led to referrals for consultations with the psychiatric emergency department (*n* = 5,174, 67.8 %). Of those who were referred for psychiatric consultation, a review of the medical records revealed that 62.3 % (*n* = 3,223) did not regularly attend their scheduled appointments as outpatients. The average wait time was 15.84 min (S.D. = 26.04), the average duration of the consultation was 57.74 min (SD = 37.34), and the mean length of stay at the Continuous Psychiatric Care Department was 70.41 min (SD = 44.956). Figure 2 shows the flow chart for both the descriptive and validation phases, as well the included and excluded patients.

### Descriptive phase outcomes: CRPT level/risk and score

Table 1 lists the outcomes of the CRPT during the descriptive phase study. The CRPT level/risk showed a majority of non-urgent (green and white, *n* = 5,668 74.28 %,) over urgent (brown, red and yellow, *n* = 1,963, 25.72 %) PEPs. Furthermore, 0.72 % (*n* = 55) were coded as brown color/risk (medical emergencies), 2.88 % (*n* = 220) were red PEP (aggressive-to-others behaviors), 22.12 % (*n* = 1,688) were yellow PEP (suicidal risk and acute psychiatric or drug-induced agitation), and the majority (59.22 %, *n* = 4,519) were green PEP (distress and low-risk PEP), Finally, 15.06 % (*n* = 1,149) were coded as white PEP (stable, no-risk PEP that requested a CPC consultation). The mean CRPT score was 24.8 (S.D. = 5.4).

**Table 1:** Outcomes of decriptive phase sample (*n* = 7,631)

Descriptive phase outcomes	Brown	Red	Yellow	Green	White	Statistics	*P*
Gender							
Male (*n*) (%)^a^	16 (0.21)	72 (0.94)	516 (6.76)	1491 (19.54)	411 (5.39)	*Χ* ^2^ = 6.86	0.143
Female (%)^a^	39 (0.51)	148 (1.94)	1172 (15.36)	3028 (39.68)	738 (9.67)		
Rate male:female	1:2.5	1:2.1	1:2.3	1:2.0	1:1.8		
Mean age (years) (SD)	47.03 (21.92)	37.04 (15.78)	34.02 (13.33)	37.98 (14.68)	38.89 16.26)	*F* = 31.38	0.0001**
Age range	15–87	13–108	13–84	13–99	12–90		
CRPT score intervals	1–9	5–16	14–23	22–31	25–32		
CRPT mean score (SD)	4.01 (1.6)	12.89 (2.5)	19.17 (3.06)	25.02 (3.1)	29.12 (2.1)	*F* = 3607	0.0001**
CRPT under triage (*n*) (%)^b^	-	16^c^ (0.21)^c^	47 (0.62)	45 (0.59)	47 (0.62)		
CRPT over triage (*n*) (%)^b^	3 (0.04)	22 (0.29)	106 (1.39)	38 (0.49)	-		
Suicide risk							
No suicidal risk (%)^b^	46 (83.63)	101 (45.91)	1179 (69.85)	4406 (97.49)	1119 (97.39)	*Χ* ^2^ = 1860	0.001*
No-suicide commitment (%)^b^	0 (0)	3 (1.36)	5 (0.29)	28 (0.62)	17 (1.48)		
Suicide thoughts (%)^b^	4 (7.27)	46 (20.91)	319 (18.89)	65 (1.44)	11 (0.96)		
Suicide threats (%)^b^	0 (0)	11 (5.00)	55 (3.26)	5 (0.11)	0 (0.0)		
Suicide attempts (%)^b^	5 (9.09)	59 (26.82)	130 (7.7)	15 (0.33)	2 (0.17)		
Reference after emergency consultation							
Urgent discharge to other hospital (%)^b^	52 (94.54)	131 (59.54)	545 (32.29)	260 (5.75)	63 (5.48)	*Χ* ^2^ = 2545	0.001*
Admitted (%)^b^	0 (0)	66 (30.00)	242 (14.34)	45 (0.99)	5 (0.99)		
Discharge to outpatient treatment (%)^b^	3 (5.46)	20 (9.09)	875 (51.83)	4028 (89.13)	1010 (87.90)		
Discharge to comunitary care (%)^b^	0 (0)	3 (1.36)	26 (1.54)	186 (4.12)	71 (6.18)		

Each PEP was broken down into one of five possible CRPT Color/Risk. Each PEP was also broken down into one score number from thirty two posible PEP. Undertriage were the PEP sorted as non urgent while were discharged as urgent PEP. Overtriage were the PEP sorted as urgent while were discharged as non urgent. PEP = Psychiatric Emergency Presentation. CPC = Continous Psychiatric Care Department (Prev. Emergency Department). CRPT: Color-Risk Psychiatric Triage

^a^Cumulative percentage of overall sample

^b^Percentage of each CRPT level/risk

^c^Undertriage subjects of Red CRPT level were sorted as a PEP with a Discharge to Medical Emergencies. Both medical and Pychiatric emergencies were considered as first priority with immediate intervention

*Significance at 0.05 level

**Significance at 0.01 level

The mean CRPT score increased, while the CRPT color/risk decreased (e.g., brown color/risk mean CRPT score was 4.01 (SD = 1.6) and white PEP mean CRPT score was 29.12 (SD = 2.1). When comparing the CRPT color/risk and the CRPT score using one-way analysis of variance (ANOVA), we found significant differences within and between groups (*F* = 3,607.25, *p* = 0.0001). Patients with medical emergencies were older than those with any type of PEP (47.03 vs. 38.89 years old, *F* = 31.38, *p* = 0.0001), while there was no age difference between patients with urgent or non-urgent PEP. The CPRT color/risk and the CRPT score were highly correlated (*r* = 0.79, *p* < 0.0001). We found that the ratio of male to female PEP was lower for urgent PEP and higher for non-urgent PEP, although this difference was not statistically significant (*Χ*^2^ = 6.86, df = 4, *p* = 0.143, ns). Both under- and over-triage of the CRPT represented less than 5 % of the overall sample.

### Validity phase outcomes: correlation between CRPT level/risk and CRPT scores

To test the concurrent and predictive validity of CRPT, three hundred patients from the descriptive phase were randomly selected to receive further and simultaneous assessments while they were undergoing general assessment by a psychiatrist or psychiatry resident from the Continuous Psychiatric Care Department staff. As shown in Fig. 2, the final validity phase included 158 subjects.

Analysis with one-way ANOVA demonstrated statistically significant differences both within and between the CRPT score and color/risk during the validity phases (*F* = 143.326, df = 4, *p* < 0,0001). Correlations between the CRPT_1_ color/risk and CRPT_1-2_ scores were highly significant (r_color1-score1_ = 0.835 and r_color1-score2_ = 0.832, *p* < 0.0001). Conversely, correlations between the CRPT_2_ color/risk and both the CRPT_1_ color/risk and score decreased but remained statistically significant (r_color2-color1_ = 0.525 and r_color2-score1_ = 0.575, *p* < 0.0001). The highest correlations were found between both CRPT_1-2_ scores (r_score1-score2_ = 0.982, *p* < 0.0001). Table 2 shows the correlations between each domain of CTRS^22^ and CRPT. There were some disparities between the CRPT_1_ and CRPT_2_ color/risk when they were correlated with discharge (r_color1-discharge_ = 0.449 and r_color2-discharge_ =0.248, *p* < 0.0001) and suicide risk (r_color1-suicide-risk_ = -0.353 vs. r_color2-suicide-risk_ = -0.160, *p* < 0.0001).

**Table 2:** Second phase outcomes: validation of CRPT (*n* = 158)

Second phase outcomes concurrent validity^a^	CRPT_1_ color/risk	CRPT_1_ score	CRPT_2_ color/risk	CRPT_2_ score	CTRS dangerousness^b^	CTRS support	CTRS cooperativeness	Total CTRS	Discharge	Suicide risk
CRPT_1_ color/risk		.835	.525	.832	.635	.476	.499	.646	.499	-.353
CRPT_1_ score	.835		.575	.982	.683	.542	.533	.704	.437	-.371
CRPT_2_ color/risk	.525	.575		.556	.402	.367	.375	.462	.248	-1.60
CRPT_2_ score	.832	.982	.556		.703	.561	.544	.732	.445	-.368
CTRS dangerousness	.635	.683	.402	.703		.636	.455	.834	.393	-.528
CTRS support	.476	.542	.367	.561	.636		.533	.845	.311	-.420
CTRS cooperativeness	.499	.533	.375	.544	.455	.533		.814	.446	-.394
Total CTRS	.646	.704	.462	.732	.834	.845	.814		.470	-.528
Predictive validity										
Discharge	.449	.437	.248	.445	.393	.311	.446	.470		-.494
Suicide risk	-.353	-.371	-.160	-.368	-.528	-.420	-,394	-.528	-.494	

It’s shown each Spearman’s rho coefficient of standardized CRPT assessment (CRPT_1_) with a simultaneous Color-Risk Psyhciatric Triage (CRPT_2_) and a Crisis Triage Rating Scale (CTRS) assessment, which were assessed by a researcher. CRTS scores was broken down by CTRS domains (Dangerousness, Support and Cooperativeness), and Overall CTRS score (3-15). In order to test predictive validity, discharge and suicidal risk were correlated with each CRPT and CRTS level/risk. CRPT: Color-Risk Psychiatric Triage. CTRS: Crisis and Triage Rating Scale (Bengersdolf et al, 1984)

^a^All correlations had <0.0001 of statistical significance

^b^Due to its risk inherent evaluation, Dangerousness was considered the most significative subdomain of CTRS for the measurement of concurrent validity of CRPT

### Validity phase outcomes: correlations between CRPT and CTRS

#### CTRS correlations

Overall, the CTRS^22^ score showed high, statistically significant correlations between each of the three domains (r_CTRSoverall-CTRSdangerousness_ = 0.834; r_CTRSoverall-CTRSsupport_ = 0.845; r_CTRSoverall-CTRScooperativenesst_ = 0.814, *p* < 0.0001). The CTRS inter-domain correlations were less than the CTRS overall score, but remained statistically significant, with the highest correlation observed for the CTRS domain of dangerousness (r_dangerousness-support_ = 0.636; r_dangerousness-cooperativeness_ = 0.455, r_support-cooperativeness_ = 0.533_,_*p* < 0.0001).

### Concurrent validity of CRPT

We found high correlations between the CTRS^22^ overall score and CRPT color/risk (r_CTRSoverall-CRPTcolor_ = 0.646, *p* < 0.0001). This correlation was greatest between the CTRS overall score and the CRPT score (r_CTRSoverall-CRPTscore_ = 0.732, *p* < 0.0001).

### Predictive validity of CRPT and CTRS

The correlations between the CRPT and CTRS^22^ final variables of discharge and suicide risk at the end of the emergency consultation were statistically significant, although not as high as the concurrent validity. In particular, we found that CTRS and CRPT showed similar predictive validity when correlated with discharge variables (r_CRPT-discharge_ = 0.449 and r_CTRS-discharge_ = 0.470, *p* < 0.0001). However, CTRS showed a higher correlation than CRPT when correlated with suicidal risk variables (r_CTRS-suicide_ = -0.528 and r_CRPT-suicide_ = -0.368, *p* < 0.0001). Table 2 shows the correlations of predictive validity for both CRPT and CTRS.

### CRPT reliability

The Intra Class Correlation (ICC) of the individual measures of the CRPT score was 0.982 (95 % CI: 0.975–0.987), and the ICC of the average measures was 0.991 (95 % CI: 0.987–0.993); these outcomes were statistically significant (*F* = 108.243, *p* < 0.0001). The ICC of individual measures for the CRPT color/risk was 0.525 (95 % CI: 0.15–0.540) and 0.575 for the average measures (95 % CI: 0.546–0.582). We assume this outcomes are exchangeable as ICC outcomes were excellent, specially those related with CRPT Score. Inter-rater reliability was also measured: Cohen’s Kappa for CRPT Score was good (*kappa* = 0.894), however, testing inter-rater reliability of CRPT color/risk showed quite lesser Cohen’s Kappa (*kappa* = 0.277). This was consistent with ICC outcomes, showing that reliability was greater for CRPT score than for CRPT color/risk.

## Discussion

Following implementation and application of the CRPT in the CPC department, we found the CRPT to be a helpful tool for sorting PEP by actual or potential risk. Hopefully, objective PEP classifications may solve certain problems such as frequent disagreements about the urgency of care in psychiatric patients, lack of objective and uniform definitions of emergencies and uncomfortable feelings about treating psychiatric disorders due to a lack of standardized procedures [24]. Furthermore, implementing a more structured PEP classification may also reduce the stigmatization of psychiatric disorders and enhance access to health care services for patients experiencing a psychiatric crisis [25].

Color/risk proportions showed that there was a marked disproportion of non-urgent vs. urgent PEP, with an average ratio of 3:1. Reasons for requesting attendance for non-urgent presentations in the ED include not having a regular healthcare provider, being able to receive care on the same day and the convenience of access to medical care around the clock [26]. Furthermore, the high proportion of non-urgent consultations in the ED is commonly attributed to the expectation that emergency services are equipped to solve a host of problems irrespective of their urgency [27]. Our perspective is that specific educational programs, designed to enhance outpatient treatment programs and to motivate patients to seek outpatient services, can decrease the number of non-urgent PEP. The ED may well be the precise location for beginning such interventions at the moment that emergency staff are involved in identifying non-urgent presentations during the triage assessment.

The CRPT assessment was conducted by psychiatrists or psychiatry residents, both members of the ED staff (CRPT_1_) and research staff (CRPT_2_). Although nurses are the most common mental health professionals involved in triage assessments in the EDs, there have been no reported differences in the positive predictive value of assessments performed by nurses compared to psychiatrists [28]. It is widely recommended that the first contact during triage be made with a medical provider [29]. Further research is needed to determine the reliability of the CRPT assessment between psychiatrists or psychiatry nurses. Nonetheless, we agree that the main factor underlying successful triage assessments is actually the environment rather than the professional conducting the assessment [30].

Evaluation of the CRPT color/risk demonstrated that green PEPs comprised the majority of presentations from the census sample. Clearly, most of the green-sorted PEP were not in severe distress and therefore were able to wait more than one hour without any problem. Our “over-green” outcomes, a reflection of the triaging of non-urgent presentations, could lead to many quality issues at the Continuous Psychiatric Care Department such as overcrowding, “left without seen” and return visits [31–33]. One potential solution may involve sorting the least severe conditions as ‘no-risk’ PEP (white color/risk), especially during a period of ED overcrowding. Educating the emergency staff is another potential solution for reducing the problem of over-triage [34]. For example, it might be easier to educate those patients with white color/risk presentations about the importance of compliance with outpatient appointments and pharmacologic treatments.

Female patients were twice as likely to receive a Continuous Psychiatric Care Department consultation than their male counterparts and were principally diagnosed with affective disorders. This finding is consistent with the greater prevalence of major depressive disorders among women [35] and the greater compliance with antidepressant treatment in white women than men [36]. In our results, men showed a higher proportion of non-urgent PEPs than women, which is inconsistent with reports showing that unmarried, less educated men present with more severe mental disorders to EDs [37]. The lower proportion of urgent PEPs among male patients in our study may be due to a higher rate of treatment refusal among males in emergency settings where the patient’s right to refuse treatment is often respected [38].

We found that 2.64 % of the descriptive phase sample was under-triaged, whereas 2.21 % was over-triaged. Although we assume that these outcomes support the use of CRPT as a valid instrument to sort psychiatric emergencies, we did not obtain information about the current state of validity and safety standards in mental triage scales. Reports that include the validity and reliability of current medical triages, such as the Manchester Triage Scale and the Canadian Triage and Acuity Scale, are limited and provide insufficient data [39]. Therefore, additional research is needed to evaluate the quality and safety standards of current mental health triage scales, including the CRPT.

CRPT showed concurrent validity with the CTRS^22^ total score, with higher correlations with the CRPT score than the CRPT color/risk. This result was perhaps due to the large number of specific categories available in the CRPT score compared to the CRPT color/risk, namely 32 possibilities vs. 5, respectively. At the time of this study, the CRPT had only recently been implemented in the CPC department and had undergone only a few standardization sessions, which conceivably led to variations in documentation by the emergency staff [40]. We found that combining both the color/risk and the score when assessing the emergency priority (e.g., “yellow-nineteen”) improved understanding among clinicians about the outcome of the CRPT and provided a more systematic classification of PEP through the use of simplified, codified categories. The resultant data may facilitate the linking of different risk levels of PEP with specific interventions or actions algorithms in EDs [41]. Thus, structured systems with clear algorithms for identifying and treating PEP could improve the comfort level of emergency room physicians in treating psychiatric patients [42].

CTRS^22^ global and inter-domain correlations were higher in the domain of “dangerousness” compared to “cooperativeness” and “social support”. This is likely because, with risk defined as the main field of psychiatric emergency classification, “cooperativeness” and “social support” serve as admission predictors rather than indicators of PEP risk.

Predictive validity outcomes for both the CRPT and CTRS^22^ were statistically significant, although the correlations were not as high as the concurrent validity outcomes. It is important to remember that the primary aim of triage scales and procedures is to provide a quick assessment to sort actual or potential danger to life or functioning and to assign a maximum wait time for the first intervention. As such, triage scales and procedures are not a substitute for clinical evaluation in the ED. In critical conditions, the risk level of the PEP may change due to the presence or absence of specific interventions. This is consistent with theories about risk being a dimensional continuum [43] and may explain why some of the urgent-sorted PEPs during triage assessment were not admitted or referred at the end of emergency consultation, as some emergencies may improve due to certain interventions during ED stay. Indeed, some studies have demonstrated that crisis intervention is associated with changes in specific treatments and even costs [44]. In other words, triage scales and instruments are helpful for sorting the condition of the user *at the point of arrival in the emergency department*, but they do not properly predict positive or negative responses to a wide range of possible interventions, such as crisis intervention, after the triage assessment.

This study had several limitations. First, the CRPT was only implemented and applied in a single psychiatric emergency department in Mexico, whose users usually present to ED voluntarily. This may have biased the outcomes, especially those involving involuntary consultations, which had to be excluded due to our inclusion criteria. CRPT external validity therefore needs to be tested in other ED, both in psychiatric and general hospitals. Another limitation of this study was the transversal patient sample, without a pre-test evaluation, which prevented assessment of the real impact on specific baseline quality indicators. CRPT outcomes also need to be studied in different populations, as our study was limited to Spanish-speaking researchers and users. Further, because the census sample population was a non-probabilistic sample, it was not representative of the general population. Another limitation is that the CRPT was designed to sort PEP of adult users, thereby omitting children and adolescent psychiatric emergencies. Finally, the CRPT does not consider special vulnerable population such as immigrants, violence victims or pregnant women, who would always be a priority regardless of their current color/risk or PEP score. In spite of these limitations, we maintain that the five-color representation of psychiatric emergencies may provide a more concise classification of PEP. As a result, this approach may enhance awareness of PEP by the ED staff from both psychiatric and general hospitals, leading to fewer delays in attendance.

## Conclusions

Our results support the use of CRPT as a novel emergency psychiatry assessment tool and dimensional color/risk and score instrument. Leading risk is the most important factor for sorting PEP priority of attendance. This approach considers actual or potential risk, rather than severity, as the main factor for sorting PEP, which improves upon the current approach to emergency classification that is mainly based on the criterion of severity. Regardless of the triage procedure, emergency assessments should no longer classify PEP as “not real emergencies.”

## Additional file

Additional file 1:
**Color Risk Psychiatric Triage.** Assessment of one from 5 Color-Risk Levels and one from 32 PEP Score. (PDF 80 kb)

## Abbreviations

CRPT: Color/Risk Psychiatric Triage
CTRS: Crisis Triage Rating Scale
ED: Emergency Department
ICC: Intra-Class Correlation Coefficient
PES: Psychiatric Emergency Service
