# Supplementary material for: Validity and reliability of a novel Color-Risk Psychiatric Triage in a psychiatric emergency department
Source: BMC Psychiatry. 2016 Feb 10;16:30. doi: 10.1186/s12888-016-0727-7 (PMC4748451; doi:10.1186/s12888-016-0727-7)
Supplement: Additional file 1: — Color Risk Psychiatric Triage. Assessment of one from 5 Color-Risk Levels and one from 32 PEP Score. (PDF 80 kb) [file 12888_2016_727_MOESM1_ESM.pdf]

COLOR-RISK PSYCHIATRIC TRIAGE

User´s Name:____ Date and Hour:____________________

Instructions: There are described ahead some Psychiatric Emergency Presentations (PEP). Assess directly or indirectly each PEP in progressive order. Each statement provides guidance for its evaluation. The evaluator has to write an "X" to sort the most risky, acute and severe color/risk level (one from five), and also has to circle a single number code which also represents a score, which best describes the actual PEP of the patient (one from thirty two). Once sorted a certain risk/color and score, interview has to be stopped at once, and evaluator has to notify outcomes to medical staff, which has to priorize attendance of high risk over low risk PEP.

FIVE COLOR/RISK LEVELS AND FIVE D'S MNEMONICS				
**1: Brown Medical Emergency**	**2: Red**<br>**High Risk PEP**	**3: Yellow**<br>**Middle Risk PEP**	**4: Green**<br>**Mild Risk PEP**	**5: White**<br>**No Risk PEP**
Delirium, Disease	Drug, Danger (Others)	Danger-Self; Disturbing	Demand, Distress	Delayed, Detached

	**NEED OF ADVANCED LIFE SUPPORT**
**01**	Unconscious patient with immediate need of Circulation, Airway and Breath Support
**02**	Confused patient, with immediate need of Circulation, Airway and Breath Support
	**ACUTE AND COMPLICATED MEDICAL CONDITIONS**
**03**	Recent neuropsychiatric symptoms with cognitive and/or motor impairment of rapid onset
**04**	Fluctuation attention disturbance with altered or unknown vital signs or laboratory outcomes
**05**	Active convulsive or epileptic features with or without treatment
**06**	Structural or functional unstability due to physical or chemical self-induced damage
	**ACUTE SUBSTANCE INTOXICATION OR WITHDRAWAL**
**07**	Substance intoxication or withdrawal with inconscious or confused mental state
**08**	Substance intoxication or withdrawal with autonomic symptoms
**09**	Substance intoxication or withdrawal without autonomic symptoms
	**ACUTE AGRESSIVE BEHAVIOR TO OTHERS**
**10**	Hits or tries to hit others with a weapon or an object
**11**	Hits or tries to hit others with own body
**12**	Threats others with an imminent pshysical agression with no hitting
**13**	Yells, Insults or curses others with no threating nor hitting
**14**	Hostile, irritable, defiant, or combative behavior, with no yelling, threating or hitting
	**SUICIDE THOUGHT, THREAT OR SUICIDE BEHAVIOR**
**15**	Current suicidal or self-injure behaviors during assessment
**16**	Current suicidal intentions during assessment
**17**	Suicide attempts or threats in the past week
**18**	Structured suicidal thoughts in the past week
	**ACUTE AND SEVERE AGITATION WITH NO AGRESSIVE BEHAVIOR**
**19**	Psychotic/Maniac Agitation: Suspicious, hallucinated, expansive and/or overactive behavior
**20**	Depressive/Anxious Agitation: Cries and/or presents severe anxious agitation
**21**	Disociative/Conversive Agitation: Agitation due to psychological Symptoms with potential risky behaviors
**22**	Dissociative/Conversive Agitation: Agitation due to psychological Symptoms with no potential risky behaviors
	**DRUG-INDUCED AGITATION OR DISTRESS**
**23**	At least one drug-induced symptom, with altered vital signs (Must be aware about body temperature)
**24**	At least one drug-induced symptom which provoques partial or general motor disability
**25**	At least one drug-induced symptom which provoques restlessness or distress
	**SPECIAL EXPECTATIVES AND NEGATIVISM**
**26**	Demands to get special attention or imposes her/his own procedures
**27**	Actively or passively rejects to receive attention without other risk behaviors
	**SUBJECTIVE PSYCHIATRIC EMERGENCY PRESENTATION**
**28**	Subjective Psychiatric Emergency with mainly physical symptoms
**29**	Subjective Psychiatric Emergency with mainly emotional symptoms
	**SEEKING ATTENTION DUE TO PERSONAL CONDITION OR DETACHED BEHAVIOR**
**30**	Personal Reasons that cuts off regular outpatient treatment
**31**	Economical Reasons that cuts off regular outpatient treatment
**32**	Schedule Reasons that cuts off regular outpatient treatment

Color/Risk:________________ **Score:__________________**

(Level and color of most acute and risky PEP, 1 to 5) (Number/Code of most acute and risky PEP, 1 to 32)

Original Version of Color-Risk Psychiatric Triage created by Molina-Lopez A MD, MSc ©2008
